# Phenotype-Genotype Correlations in Mouse Models of Amelogenesis Imperfecta Caused by *Amelx* and *Enam* Mutations

**DOI:** 10.1159/000336440

**Published:** 2012-06-28

**Authors:** Thomas Liam Coxon, Alan Henry Brook, Martin John Barron, Richard Nigel Smith

**Affiliations:** ^a^School of Dentistry, Faculty of Health and Life Sciences, Dental Hospital, Liverpool, UK; ^b^Faculty of Life Sciences and School of Dentistry, Manchester Academic Health Sciences Centre, University of Manchester, Manchester, UK

**Keywords:** Enamel, Mineralization, Amelogenesis imperfecta, Mouse model, Phenotype-genotype

## Abstract

Mutations in human and in mouse orthologous genes *Amelx* and *Enam* result in a diverse range of enamel defects. In this study we aimed to investigate the phenotype-genotype correlation between the mutants and the wild-type controls in mouse models of amelogenesis imperfecta using novel measurement approaches. Ten hemi-mandibles and incisors were dissected from each group of *Amelx*^WT^, *Amelx*^X/Y64H^, *Amelx*^Y/Y64H^, *Amelx*^Y64H/Y64H^, and *Enam*^WT^, *Enam*^Rgsc395^ heterozygous and *Enam*^Rgsc395^ homozygous mice. Their macro-morphology, colour and micro-topography were assessed using bespoke 2D and 3D image analysis systems and customized colour and whiteness algorithms. The novel methods identified significant differences (p ≤ 0.05) between the *Amelx* groups for mandible and incisor size and enamel colour and between the *Enam* groups for incisor size and enamel colour. The *Amelx*^WT^ mice had the largest mandibles and incisors, followed in descending order of size by the *Amelx*^X/Y64H^, *Amelx*^Y/Y64H^ and *Amelx*^Y64H/Y64H^ mice. Within the *Enam* groups the *Enam*^WT^ incisors were largest and the *Enam*^Rgsc395^ heterozygous mice were smallest. The effect on tooth morphology was also reflected by the severity of the enamel defects in the colour and whiteness assessment. Amelogenin affected mandible morphology and incisor enamel formation, while enamelin only affected incisors, supporting the multifunctional role of amelogenin. The enamelin mutation was associated with earlier forming enamel defects. The study supported the critical involvement of amelogenin and enamelin in enamel mineralization.

## Introduction

Many genes underlying normal and abnormal dental development have been identified using studies on mouse models [Thesleff, 2006; Fleischmannova et al., 2008]. However, the underlying pathogenesis of the clinically and genetically heterogeneous group of enamel defects, amelogenesis imperfecta (AI), requires further investigation [Wright et al., 2009]. Correlating accurately defined phenotypes with different genotypes will contribute to understanding the effect of specific mutations responsible for AI.

### Morphometry

In humans quantitative methods for clinical phenotyping of the dentition have been developed, from hand calliper measurements to two-dimensional (2D) [Brook et al., 1986, 2005] and now three-dimensional (3D) imaging [Smith et al., 2009b]. Each new advance has enabled additional parameters to be determined and phenotyping to be enhanced.

Murine mandibles and incisors represent excellent models of complex morphological structures [Atchley and Hall, 1991], including a permanent record of all developmental stages of enamel formation, for which quantification of the effects of specific variables is valuable [Cooper and Albertson, 2008].

### Enamel Colour and Surface Roughness Assessment

The physico-optical properties of dental hard tissues reflect the normal and abnormal process of mineralization [Joiner et al., 2008]. Tooth morphology and the enamel crystal surface [Risnes, 1979; Warshawsky et al., 1987] affect light reflectance and influence colour.

Colour distribution in human incisors has been used to assess enamel in three anatomical regions – cervical, middle and incisal [Brook et al., 2007]. In mice enamel development has been separated into various different stages, including secretory, pre-eruptive and mature [Robinson et al., 1983; Smith and Nanci, 1989; Wong et al., 2000; Smith et al., 2009a].

International recommendations for the objective measurement of colour [Commission Internationale de l'Eclairage, 1986] and surface texture [International Organization for Standardization, 1996] have been calibrated for human clinical trials [Smith et al., 2008] and used to interrogate enamel surface mineralization [Zhang et al., 2000; Higham et al., 2009]. These novel methods will provide a complementary approach to reflect enamel development, structure and function.

### Amelogenesis Imperfecta

AI is a clinically and genetically heterogeneous group of inherited dental enamel defects [Witkop, 1967]. Amelogenesis is orchestrated by genetic regulation of the secretion, organization and processing of the developing enamel extracellular matrix (ECM) [Wright, 2006]. A number of mutations in the amelogenin gene *(AMELX)* [Hart et al., 2002; Kim et al., 2004] and the enamelin gene (*ENAM*) [Rajpar et al., 2001; Mardh et al., 2002] are implicated in the aetiology of types of AI in humans and mice [Wright et al., 2009].

The X-linked forms of AI (AIH1) are associated with specific mutations in the amelogenin gene (*AMELX,* OMIM ID300391) [Lau et al., 1989; Aldred et al., 1992; Salido et al., 1992]. A diverse range of AIH1 phenotypes is observed from smooth hypoplastic to hypomineralized/hypomaturation enamel [Wright et al., 2003]. Specific mutations in the enamelin gene (*ENAM,* OMIM ID606585) are associated with autosomal dominant AI (AIH2) [Rajpar et al., 2001], of which two phenotypically distinct forms are reported – smooth hypoplastic AI and local hypoplastic AI.

### Phenotyping Mouse Models

Similar enamel phenotypes have been reported for some mutant mice and humans [Gibson et al., 2001; Hart et al., 2002; Seedorf et al., 2007]. Anatomical, histological and ultrastructural assessment of mice models of AI have revealed key factors underpinning the molecular pathogenesis of AI, e.g. the disrupted secretion of enamelin interferes with initial enamel crystal formation [Masuya et al., 2005] and ameloblast cell binding and the intracellular proetin trafficking may have a mechanistic role in the failed secretion of amelogenin into the ECM [Barron et al., 2010].

However, objective morphometric measurement of mandibles and incisors, together with colour and whiteness and surface assessment of incisors, has not been described in any previous study. Quantitatively assessing the phenotype of mandibles and incisors associated with known mutations *Enam* S55I [Masuya et al., 2005] and *Amelx* Y64H [Barron et al., 2010] will aid in studying the outcome of the mutations and relating the phenotype to the genotype of these two pertinent mouse models of AI.

The aim of this study was to examine and interpret correlations between genotype and phenotype in three *Amelx* mouse groups, two *Enam* groups and their respective wild-type controls. Such correlations should provide further insight into the functions of amelogenin and enamelin during normal dental development and strengthen our understanding of aberrant enamel mineralization.

## Materials and Methods

The mice were bred and reared under identical standard conditions in accordance with the Animals (Scientific Procedures) Act, UK, 1986. All mice were euthanized at the same age (90 days) and weight (25 ± 5 g). Mice were gender matched within and between groups, and they were fed a soft diet. Mice were examined using the protocols of European Mouse Phenotyping Resource of Standardized Screens (www.empress.har.mrc.ac.uk/). Ethics approval was granted according to Wellcome programme GRO75945MA ethics reference number 06/Q0104/38. DNA was extracted from ear skin samples of each mouse used in the study. Genotyping, using these DNA samples, was performed for each mouse using oligonucleotide primers to PCR amplify the *Amelx* and *Enam* genes followed by DNA sequencing using dye primer chemistry.

For the reliability study, left and right hemi-mandibles and mandibular incisors were dissected from a mixed sex population of Charles River CD-1 wild-type mice (n = 20) (Charles River, Inc., Boston, Mass., USA). For the main study, mice containing the *Amelx* M100888 mutation (MGI ID3807977) and the *Enam* M100395 mutation (MGI ID3055582), generated at RIKEN GSC, Tokyo, Japan, in their large-scale ENU mutagenesis programme (www.brc.riken.jp/lab/gsc/mouse/), were used. The left and right hemi-mandibles and mandibular incisors were extracted from the *Amelx* wild-type (*Amelx*^WT^), *Amelx* heterozygous (*Amelx*^X/Y64H^), hemizygous (*Amelx*^Y/Y64H^) and homozygous (*Amelx*^Y64H/Y64H^) genotype groups, and from the *Enam*^Rgsc395^ wild-type (*Enam*^WT^), *Enam*^Rgsc395^ heterozygous and *Enam*^Rgsc395^ homozygous genotype groups (n = 5 in each group). The *Amelx*^WT^ and *Enam*^WT^ mice were littermate controls.

### Mandible and Incisor Extraction

Specimens were preserved in 10% neutral buffered formalin and washed in phosphate-buffered saline and distilled water before examination. Micro-dissection of hemi-mandibles and incisors was carried out under a dissection microscope (Bresser, Meade Instruments Corp., Irvine, Calif., USA). Incisors were removed after hemi-mandible imaging with care to avoid mechanical damage to surface enamel. Any incisors that were seen to be damaged at the microscopic level were discarded from the study, according to strict visual and tactile criteria, e.g. scalpel marks, and so did not interfere with roughness measurements. Specimens were kept on ice to minimize any temperature effects or dehydration during imaging.

### Imaging

Standardized 2D images were taken with a 13.5-megapixel Kodak DCS Pro SLR/n (Eastman Kodak Company, Geneva, Switzerland) digital camera using an established image analysis system (IAS) [Brook et al., 2005]. An MP-E 65-mm F2.8 1–5× Macro Photo Lens (Sigma Corp., Kanagawa, Japan) was used. Each image contained an 11.0-mm scale and was calibrated individually using Image Pro Plus version 5.1 software (Media Cyberenetics, Inc., Bethesda, Md., USA).

The 3D IAS consisted of a customized non-contact surface profilometer (Scantron ProScan 2000; ScanTron Industrial Products Ltd., Taunton, UK) with a high systematic resolution (1.0 μm). The device was modified to rotate incisors through 360° and 3D models were constructed using SolidWorks Premium 2008 software (Dassault SolidWorks, Waltham, Mass., USA).

The colour and whiteness images of the incisor labial surface were captured to contain the whole enamel surface. Images were automatically calibrated against a spectrophotometrically assessed standardized white tile (British Ceramic Research Association). Polarized images avoided interference from surface reflections.

### Morphometric Assessment

**Fig. 1:**
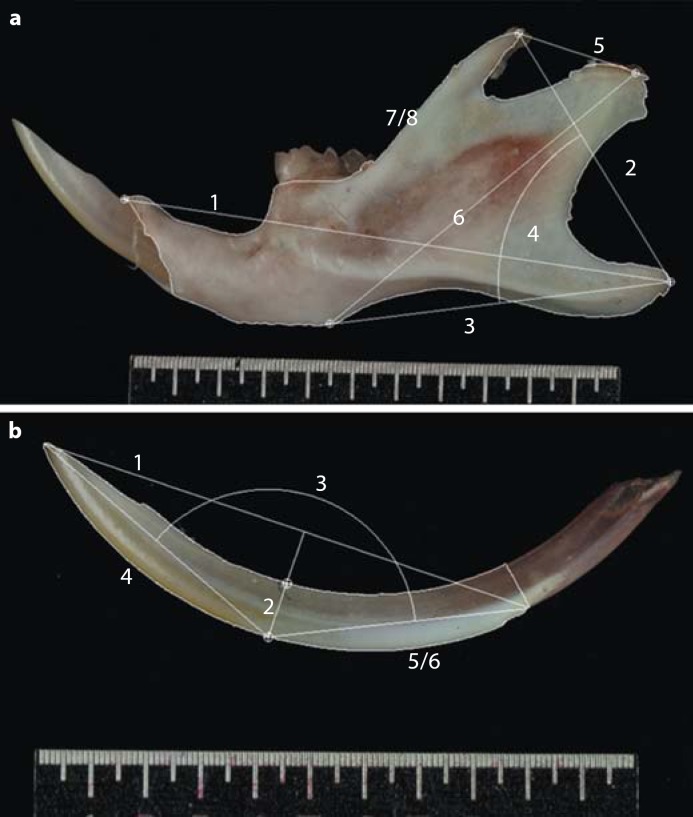
Hemi-mandible and mandibular incisor 2D morphological measurements. **a** Hemi-mandible 2D morphometric variables: (1) overall length (mm); (2) ascending height (mm); (3) basal length (mm); (4) mandible angle (degrees); (5) coronoid-coronoid (mm); (6) diagonal length (mm); (7) mandible area (mm^2^), and (8) mandible perimeter (mm). **b** Mandibular incisor 2D morphometric variables: (1) overall length (mm); (2) angle of curvature (degrees); (3) width at midpoint (mm); (4) labial length (mm); (5) incisor perimeter (mm), and (6) incisor area (mm^2^). Left hemi-mandible and mandibular incisor shown from the buccal view. Scale bar = 11.0 mm.

**Fig. 2:**
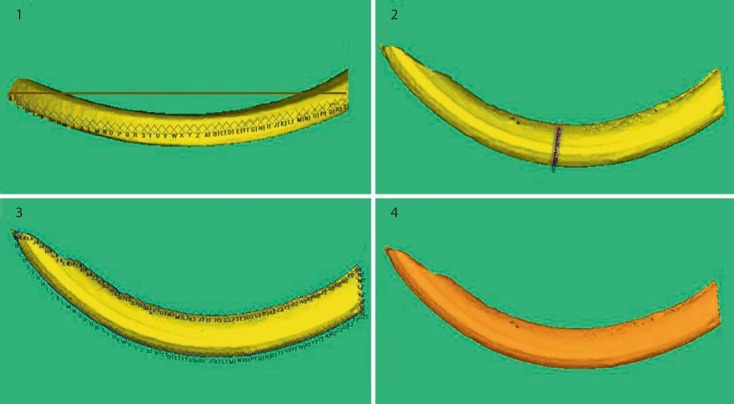
A selection of mandibular incisor 3D morphological measurements: (1) the line of x's demonstrate the path of the actual labial-length, whilst the straight line shows the projected 2D length; (2) actual width-at-midpoint (mm); (3) actual perimeter (mm); (4) marked surface-area (mm^2^).

After 2D image acquisition the specimens were automatically outlined by the Image Pro Plus software and morphological measurements obtained from the hemi-mandibles (fig. 1a) and incisors (fig. 1b). All 3D morphological measurements were obtained using Cloud 3D surface viewer software (Dr. Robin Richards, Westcott Road, London) that enabled both projected and actual linear measurements (fig. 2).

### Enamel Colour and Whiteness, and Surface Roughness Assessment

**Fig. 3:**
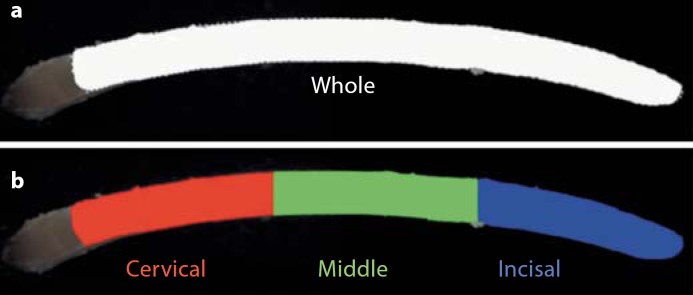
Mandibular incisor calibrated colour and whiteness. **a** Whole enamel surface region outlined. **b** Automatically separated cervical, middle and incisal anatomical surface region developmental stages. An automated algorithm calculated CIE L, A and B, and WI colour space values for each of the four regions of interest. Polarized images removed interference from surface reflections. Left mandibular incisor shown from the labial view.

For colour and whiteness assessment, incisors were held in a customized holder with the proximal end fixed in black modelling clay. The buccal surface enamel was imaged from the labial view (fig. 3). At the proximal end of each incisor a distinctive colour and surface texture change was used as a readily identifiable anatomical landmark feature. This was consistently observed in the 2D morphometric images, the colour and whiteness images and in the 3D images. This white opaque boundary was previously reported [Robinson et al., 1983; Smith and Nanci, 1989].

Images were opened in customized Adobe Photoshop software CS2 version 9 (Adobe Systems, Inc., San Jose, Calif., USA) and the ‘Magnetic Lasso Tool’ feature was used to objectively trace the observable incisor perimeter that encompassed the whole labial enamel surface. Using customized hotkeys, red, green and blue colour channel outputs were automatically derived either from the whole enamel surface area [(i) whole] or from one of the three regions that were separated equidistantly into (ii) cervical, (iii) middle and (iv) incisal. Simultaneously, a Microsoft Excel (Microsoft Corp., Albuquerque, N. Mex., USA) algorithm used the red, green and blue outputs to calculate calibrated CIE L, A and B (L = lightness, A = green/red, B = yellow/blue) colour space and WI (WI = whiteness) values, where L = 0 yielded black and L = 100 yielded white [Smith et al., 2008].

These additional three regions were chosen as they provided a greater degree of analysis than just using the entire surface as a whole. The authors appreciate that the actual underlying histological regions differ; however, they are not visible at the tooth surface to use as a method guide. The software and algorithm minimized human subjective input and error, were highly reproducible, objective and practical and expedited data collection efficiently.

**Fig. 4:**
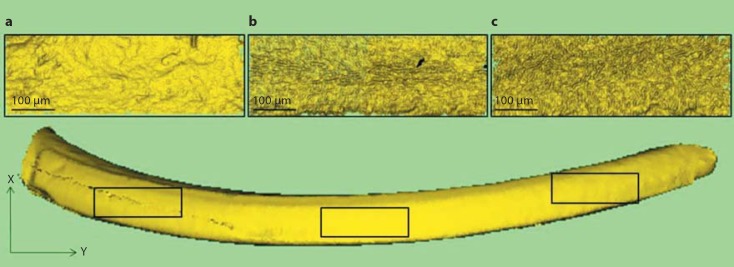
Mandibular incisor surface roughness assessment. Labial surface enamel regions, cervical (**a**), middle (**b**) and incisal (**c**), represent the stages of enamel formation, respectively. Right mandibular incisor shown from the labial view. Left: proximal end, right: distal tip.

A surface roughness measurement was taken in each of the three specific enamel surface regions in a 3D image from each experimental group (sample size n = 1) using ProScan 2000 software (ScanTron Industrial Products Ltd.) (fig. 4). A 200 × 500 μm area was selected equidistantly along the longitudinal axis of the incisor to minimize subjectivity. The surface roughness measurement quantified enamel surface texture, as distinct from form or waviness components [International Organization for Standardization, 1996].

### Reliability and Validation

Measurements were taken by two independent operators. The initial and repeat imaging and measurements were carried out on different days. No repeats were obtained for surface roughness. Using SPSS (SPSS, Inc., Chicago, Ill., USA), intra-class correlation coefficient (ICC), Pearson's correlation coefficient (PCC) and repeated measures t tests were used to determine method reliability and agreement [Fleiss, 1986]. Using MedCalc (MedCalc bvba, Mariakerke, Belgium) Bland-Altman plots visualized limits of agreement and bias.

### Phenotypic Comparison

One operator implemented the phenotypic comparisons between the four *Amelx* genotype groups and the three *Enam* genotype groups. Percentage values were obtained by dividing the number of significantly different variables by the total number of variables to indicate the relative number of significant differences found using each variable. Descriptive statistics provided the mean, mean difference, standard error and 95% confidence intervals. Bonferroni's corrected one-way ANOVA multiple comparisons (p = 0.002) and post hoc Tukey's honestly significant difference tests (p = 0.05) were used to identify significant phenotype variation between groups. Significant differences (p = 0.05) observed before the robust Bonferroni correction are also detailed because of the varying degrees of independence of the variables.

## Results

### Reliability and Validation

Intra-operator repeatability (ICC ≥0.75) and inter-operator reproducibility (ICC ≥0.77) were predominantly substantial to excellent for all 2D and 3D morphometric variables according to the classification of Donner and Eliasziw [1987]. A similarly excellent (ICC ≥0.96) intra-operator repeatability was demonstrated for colour and whiteness assessment across all of the enamel surface regions.

The 2D and 3D methods showed significant (p ≤ 0.01) method agreement (PCC 0.710–0.999) [Rodgers and Nicewander, 1988]. Repeated measures t tests showed no significant differences (p ≥ 0.01) between measurements, except for the width-at-midpoint variable. The Bland-Altman plots used to assess limits of agreement and bias gave satisfactory results [Bland and Altman, 1986, 1999].

### Phenotypic Comparison

#### *Amelx* Groups

Twenty-five percent of mandible and 82% of incisor 2D variables showed significant differences (p ≤ 0.05) between one or more of the *Amelx* groups (table 1). The *Amelx*^WT^ group had the largest mandibles (e.g. ascending height and mandible angle) and incisors (e.g. overall length, perimeter and area), followed by the *Amelx*^X/Y64H^, then the *Amelx*^Y/Y64H^ and finally the *Amelx*^Y64H/Y64H^ groups (online suppl. fig. 1; for all online suppl. material, see www.karger.com?doi=10.1159/000336440).

Eighty-four percent of colour and whiteness variables showed significant differences (≤0.05) between the *Amelx* groups, notably between all of the groups in the incisal and whole regions (table 1). The major colour and whiteness differences between the *Amelx*^WT^ and *Amelx*^Y/Y64H^ groups and between the *Amelx*^WT^ and *Amelx*^Y64H/Y64H^ groups occurred in the lightness, yellow/blue and whiteness colour components (online suppl. fig. 1). The *Amelx*^WT^ and *Amelx*^X/Y64H^ groups’ yellow/blue values were similar and significantly higher than those of the *Amelx*^Y/Y64H^ and *Amelx*^Y64H/Y64H^ groups.

Seventy-three percent of incisor 3D variables showed significant differences (p ≤ 0.05) between one or more of the *Amelx* groups (table 1). The *Amelx*^WT^ incisors were the largest (e.g. surface area and volume), and the *Amelx*^Y64H/Y64H^ incisors were the smallest; the *Amelx*^X/Y64H^ and *Amelx*^Y/Y64H^ incisors were of an intermediate size (online suppl. fig. 2).

### Enam Groups

There were no significant differences in mandible morphometry between the *Enam* groups. Only 14% of incisor 2D variables showed significant differences (p ≤ 0.05) between the *Enam*^WT^ and *Enam*^Rgsc395^ heterozygous groups (table 2).

Forty-one percent of the colour and whiteness variables showed a statistically significant difference (≤0.05) between the *Enam* groups (table 2). Significant colour and whiteness differences occurred between the *Enam*^WT^ and *Enam*^Rgsc395^ heterozygous groups and between the *Enam*^WT^ and *Enam*^Rgsc395^ homozygous groups (online suppl. fig. 3). The average yellow/blue component of *Enam*^WT^ was higher than the *Enam*^Rgsc395^ heterozygous and the *Enam*^Rgsc395^ homozygous groups in the middle, incisal and whole regions in that order.

Eighty percent of incisor 3D variables showed significant differences (p ≤ 0.05) between the *Enam*^WT^ and the *Enam*^Rgsc395^ heterozygous mice (table 2). The *Enam*^WT^ incisors were the largest (e.g. projected overall length), followed by the *Enam*^Rgsc395^ homozygous group and then the *Enam*^Rgsc395^ heterozygous group (online suppl. fig. 3).

Regarding surface roughness assessment, *Amelx*^WT^ and *Enam*^WT^ had similar marginally higher values compared to their respective mutant groups (tables 1, 2). In all groups the enamel surface roughness increased through the cervical, middle and incisal surface regions (online suppl. fig. 2, 4).

## Discussion

Reliability was substantial to excellent for almost all variables. The 2D and 3D method agreement validated the new 3D IAS [Rodgers and Nicewander, 1988]. The complementary methods provide objective approaches to quantitative phenotypic analysis of mice mandibles and incisors that are of comparable reliability to those used for human teeth [Smith et al., 2009b]. These novel experimental approaches have considerable potential for future applications, for example small mammalian dentition and other similar murine model organisms.

The significant mandible morphological differences found between the *Amelx* groups, but not between the *Enam* groups, support a role for amelogenin in mandible development. Amelogenin is expressed in various developing structures including dental supporting tissues and during alveolar bone formation and remodelling [Gruenbaum-Cohen et al., 2008]. Our findings are consistent with its involvement in formation and growth of the mandible ramus. This supports amelogenin's role as a multifunctional protein in the craniofacial complex.

There was evidence of significant phenotypic differences between the controls and the mutant mice. These enamel mineralization defects were associated with the absence of the full length amelogenin and enamelin proteins in the developing enamel ECM [Seedorf et al., 2007; Smith et al., 2009a]. The significant macroscopic differences between the incisors, for the wild-type controls and the mutant groups, concur with the important contribution of amelogenin and enamelin in structural organization and enamel mineralization, detectable at the phenotype level.

The 2D and 3D morphological data suggested the enamel in the *Enam*^Rgsc395^ homozygous incisors was less affected than that in the *Enam*^Rgsc395^ heterozygous mutants. This contrasted with the report by Smith et al. [2009a] that indicated a more severe phenotype for the *Enam*^Rgsc395^ homozygous null mutants. However, only 14% of the 2D morphological variables were significantly different between the *Enam* wild types and the *Enam* heterozygous mice. Also, there were no significant differences between the 3D incisor morphology of the *Enam* wild types and the *Enam* homozygous, or between the *Enam* heterozygous and *Enam* homozygous mice.

The wild-type mouse incisors showed typical rodent enamel and dentine colouration, i.e. opaque white with yellow/orange/brown colouration, due to the deposition of iron pigment in the superficial layer of enamel [Halse, 1972]. However, this layer may have been disrupted during preeruptive enamel maturation leading to the observed chalky white enamel indicative of porous hypoplastic enamel of AI.

The significant differences in colour and whiteness between the *Amelx*^X/Y64H^ and *Amelx*^Y/Y64H^ groups reflect a mosaic genotype in the *Amelx*^X/Y64H^ females concordant with the expression of the mutant *Amelx* allele according to lyonization [Lyon, 1961] or X-chromosomal inactivation [Huynh and Lee, 2005]. The *Amelx*^X/Y64H^ females showed hypomineralized enamel and the *Amelx*^Y/Y64H^ males and *Amelx*^Y64H/Y64H^ females displayed thin severely hypoplastic enamel characteristic of AI [Wright, 2006].

The *Amelx*^WT^ and *Enam*^WT^ control groups both had high yellow/blue and low whiteness and lightness values in the incisal region, correlating with the contribution of both amelogenin and enamelin to the intact and normally mineralized enamel phenotype. In contrast, the mutant *Amelx* and *Enam* mice had significantly lower yellow/blue values and higher whiteness and lightness values. Smith et al. [2009a] reported flaky enamel in the *Enam* homozygous null incisors compared to the *Enam* heterozygous incisors; however, both the *Enam*^Rgsc395^ heterozygous and homozygous mice incisors displayed flaky enamel.

The site of these significant differences varied between the *Amelx* groups and the *Enam* groups: in the *Amelx* mice the differences between the groups were found in the incisal region, while in the Enam mice the differences between the groups occurred in both the middle and incisal regions. This suggests that *Enam* may have an earlier, more generalized effect on colour and whiteness than *Amelx*. The study differentiates between the overlapping enamel phenotypes of hypomineralized *Amelx*^X/Y64H^ females and severely hypoplastic *Amelx*^Y/Y64H^ males and *Amelx*^Y64H/Y64H^ females, and local hypoplastic *Enam*^Rgsc395^ *heterozygous* and *homozygous* mice, according to the two mutations, in an enamel surface region-specific manner that correlates to the distinct stages of enamel formation [Gibson et al., 2007].

The enamel surface roughness increased through the cervical, middle and incisal surface regions that represented the progressive developmental stages of enamel mineralization. However, the sample size did not allow for statistical significance to be tested and was an experimental limitation. Also, these findings contrasted with the diminishing surface roughness expected from a loss of organic matrix and an increasingly smooth crystal surface morphology as revealed by atomic force microscopy [Kirkham et al., 1998]. The incisal surface region was the only enamel surface region to have erupted into the oral cavity and be exposed to attrition or abrasion. However, all mice were maintained under identical standard conditions and fed on soft diets, which minimized the potential impact of any external environmental influences.

The increased surface roughness observed was consistent with the presence of pathological enamel, as mutations that disrupt ECM processing impair enamel mineral formation and disrupt crystal morphology [Robinson et al., 2003]. This supports the recently proposed hypothesis that intracellular protein-protein interactions involved in the secretion of amelogenin are a key mechanistic factor underpinning AIH1 [Barron et al., 2010].

Comparing these phenotypic observations in mice with the enamel defects due to *AMELX* and *ENAM* mutations in humans [Gibson et al., 2001; Hart et al., 2002] must be undertaken with care [Masuya et al., 2005; Gibson et al., 2007], noting the variation in the splicing of amelogenin, the cleavage products of enamelin, in protein function and epigenetic effects [Wright et al., 2003; Hu et al., 2007; Wright et al., 2009].

In conclusion, significant mandibular and incisor morphometric as well as colour and whiteness differences between the wild-type controls and specific mutant phenotypes were related to aberrant enamel mineralization caused by the presence of amelogenin and enamelin proteins during amelogenesis. The multifunctional role of amelogenin in mandibular development was also supported.

## Supplementary Material

Supplemental Figures

## Figures and Tables

**Table 1:** *Amelx* significant mandible and incisor morphometry, colour and whiteness and surface roughness variables

Morphometric variable	Group (n = 5)							
	*Amelx* wild-type		*Amelx* heterozygous		*Amelx* hemizygous		*Amelx* homozygous	
	buccal	lingual	buccal	lingual	buccal	lingual	buccal	lingual
Mandible								
Ascending height, mm	6.01 (0.06)	6.05 (0.09)	5.89** (0.08)	6.02** (0.11)	5.76 (0.12)	5.71 (0.16)	5.54** (0.05)	5.52** (0.06)
Mandible angle, degrees	66.22 (0.57)	65.17 (0.59)	67.26 (1.36)	66.22 (1.48)	67.84 (0.91)	62.28 (0.85)	71.88** (0.54)	70.89* (0.57)
2D incisor								
Overall length, mm	10.56 (0.34)	10.46 (0.25)	10.34* (0.21)	10.52** (0.33)	10.19* (0.21)	10.27** (0.19)	9.00* (0.10)	9.33** (0.08)
Incisor angle, degrees	117.48 (1.13)	117.32 (1.27)	116.47* (1.11)	116.45* (1.24)	119.23* (0.76)	119.50* (0.72)	134.23* (0.84)	132.12* (0.97)
Width at midpoint, mm	0.99 (0.01)	0.99 (0.01)	0.98** (0.02)	0.98* (0.01)	0.94 (0.01)	0.94** (0.01)	0.91* (0.2)	0.92** (0.02)
Perimeter, mm	25.01 (0.87)	24.88 (0.67)	25.18* (0.60)	25.09* (0.62)	24.29* (0.25)	24.25* (0.27)	20.03* (0.27)	20.73* (0.36)
Area, mm^2^	10.96 (0.51)	11.02 (0.39)	10.83* (0.36)	10.84* (0.43)	10.11* (0.15)	10.16* (0.16)	8.23* (0.08)	8.55* (0.15)
3D incisor (labial)								
Projected labial length, mm	9.83 (0.36)		9.22 (0.27)		9.10 (0.17)		8.58** (0.29)	
Actual labial length, mm	10.91 (0.47)		10.03 (0.40)		10.03 (0.180)		9.32** (0.33)	
Circumference, mm	2.96 (0.065)		2.70 (0.06)		2.81 (0.11)		2.56** (0.06)	
Total surface area, mm^2^	27.95 (0.59)		23.92 (0.78)		22.80** (0.43)		23.81** (1.39)	
Volume, mm^2^	5.37 (0.22)		4.71** (4.46)		4.35* (0.12)		4.32* (0.15)	

	Group (n = 5)							
	*Amelx* wild-type				*Amelx* heterozygous			
	cervical	middle	incisai	whole	cervical	middle	incisai	whole
Colour component								
Lightness	48.52 (1.07)	44.96 (2.36)	44.26 (1.06)	45.89 (1.48)	37.66* (2.13)	36.86* (3.17)	32.83* (2.67)	35.97* (1.59)
Red/green	−4.32 (0.31)	−4.04 (0.25)	−4.81 (0.21)	−4.41 (0.12)	−1.70 (1.36)	−0.80** (1.01)	−0.32* (0.46)	−1.08* (0.77)
Yellow/blue	3.09 (1.34)	4.50 (0.21)	11.26 (0.68)	6.05 (0.50)	6.00 (1.98)	6.48 (3.39)	8.39* (1.56)	6.80* (1.74)
Whiteness	76.68 (8.01)	67.87 (1.38)	26.06 (3.97)	58.83 (2.99)	61.34 (12.10)	58.07 (22.36)	42.18* (11.29)	56.50* (10.58)
Surface roughness (n = 1)	2.00	3.20	5.40	−	1.90	2.10	2.30	−

	*Amelx* hemizygous				*Amelx* homozygous			
	cervical	middle	incisai	whole	cervical	middle	incisai	whole
Colour component								
Lightness	39.00* (1.34)	45.73* (1.18)	40.96* (1.06)	41.63* (1.09)	39.83* (0.95)	51.70* (0.91)	54.71* (1.76)	48.45* (0.99)
Red/green	−2.67 (0.26)	−1.68** (0.33)	−0.42* (0.22)	−1.55* (0.27)	−3.29 (0.19)	−2.98 (0.41)	−4.82* (0.39)	−3.68* (0.29)
Yellow/blue	1.97 (0.91)	0.07 (0.65)	−1.15* (1.08)	0.35* (0.76)	3.23 (1.06)	−0.51 (1.12)	−1.55* (0.50)	0.42* (0.75)
Whiteness	89.00 (4.92)	97.6 (3.26)	107.45* (5.36)	97.93* (3.58)	76.11 (7.10)	96.32 (6.40)	100.94* (3.01)	92.03* (4.55)
Surface roughness (n = 1)	1.50	1.90	2.10	−	2.00	2.3	3.40	−

Mean values are shown. Values in parentheses represent the standard error. One-way ANOVA multiple comparison and post hoc tests determined * Bonferroni corrected significant differences (p ≤ 0.002) and ** significant differences (p ≤ 0.05). Right side not displayed for brevity.

**Table 2:** *Enam* significant mandible and incisor morphometry, colour and whiteness and surface roughness variables

Morphometric variable	Group (n = 5)					
	*Enam* wild-type		*Enam* homozygous		*Enam* heterozygous	
	left buccal	left lingual	left buccal	left lingual	left buccal	left lingual
2D incisor						
Incisor angle, degrees	128.5 (0.77)	128.69 (0.30)	128.88 (0.72)	128.72 (22.97)	130.03 (0.84)	130.81* (0.71)
3D incisor (labial)						
Projected labial length, mm	9.82 (0.30)		9.06 (027)		8.59** (0.19)	
Actual labial length, mm	10.69 (0.41)		10.17 (0.37)		9.26** (1.60)	
Circumference, mm	2.90 (0.09)		2.76 (0.10)		2.53** (0.05)	
Total surface area, mm^2^	26.87 (0.64)		25.66 (1.32)		22.53** (0.60)	
Volume, mm^2^	6.23 (0.55)		5.02 (0.31)		4.31** (0.25)	

	Group (n = 5)			
	*Enam* wild-type			
	cervical	middle	incisal	whole
Colour component				
Lightness	59.60 (3.30)	42.08 (2.10)	42.77 (3.12)	42.67 (2.07)
Red/green	−3.20 (0.87)	−2.69 (0.30)	−3.41 (0.93)	−3.17 (0.64)
Yellow/blue	5.17 (0.59)	6.64 (1.26)	13.33 (1.54)	8.15 (0.62)
Whiteness	65.03 (4.19)	55.94 (7.87)	13.33 (9.80)	46.55 (3.93)
Surface roughness (n = 1)	2.80	3.60	5.10	−

	*Enam* homozygous			
	cervical	middle	incisal	whole
Colour component				
Lightness	33.01* (4.41)	44.47 (2.80)	45.87 (3.06)	41.1 (2.91)
Red/green	−0.38 (1.38)	−1.86 (0.54)	−2.11 (0.91)	−1.58 (0.69)
Yellow/blue	7.06 (1.28)	2.56* (1.05)	−3.02* (0.23)	2.11* (0.63)
Whiteness	50.20 (10.29)	81.9* (6.21)	114.26* (1.31)	85.49* (4.04)
Surface roughness (n = 1)	2.40	2.80	3.50	−

	*Enam* heterozygous			
	cervical	middle	incisal	whole
Colour component				
Lightness	38.28* (2.43)	45.31 (1.06)	47.33 (1.34)	43.54 (1.06)
Red/green	−1.24 (0.97)	−2.79 (0.61)	−3.09 (0.98)	−2.41 (0.79)
Yellow/blue	5.09 (0.80)	1.97* (0.94)	2.95* (3.44)	3.30* (1.36)
Whiteness	65.61 (4.60)	84.71* (4.48)	78.41* (20.16)	77.23* (8.04)
Surface roughness (n = 1)	1.90	2.30	4.20	−

Mean values are shown. Values in parentheses represent the standard error. One-way ANOVA multiple comparisons and post hoc tests determined * Bonferroni corrected significant differences (p ≤ 0.002) and ** significant differences (p ≤ 0.05). Right side not displayed for brevity.

## Abbreviations

2D: two-dimensional
3D: three-dimensional
AI: amelogenesis imperfecta
AIH1: X-linked amelogenesis imperfecta
AIH2: autosomal dominant local hypoplastic amelogenesis imperfecta
AMELX: amelogenin human X chromosome gene
Amelx: amelogenin mouse X chromosome gene
Amelx^WT^: amelogenin wild-type
Amelx^X/Y64H^: heterozygous Y64H mutation
Amelx^Y/Y64H^: hemizygous Y64H mutation
Amelx^Y64H/Y64H^: homozygous Y64H mutation
ECM: extracellular matrix
ENAM: enamelin human gene
Enam: enamelin mouse gene
Enam^WT^: enamelin wild-type
Enam^Rgsc395^: heterozygous S55I mutation
Enam^Rgsc395^: homozygous S55I mutation
IAS: image analysis system
ICC: intra-class correlation coefficient
PCC: Pearson's correlation coefficient
