# Supplementary material for: Phenotype-Genotype Correlations in Mouse Models of Amelogenesis Imperfecta Caused by Amelx and Enam Mutations
Source: Cells Tissues Organs. 2012 Jun 28;196(5):420–30. doi: 10.1159/000336440 (PMC3718574; doi:10.1159/000336440)
Supplement: Supplementary file 1 — Supplemental Figures [file cto-0196-0420-s01.docx]

**Supplementary Material**

**Fig. S1.** *Amelx* 2D Phenotype Comparison


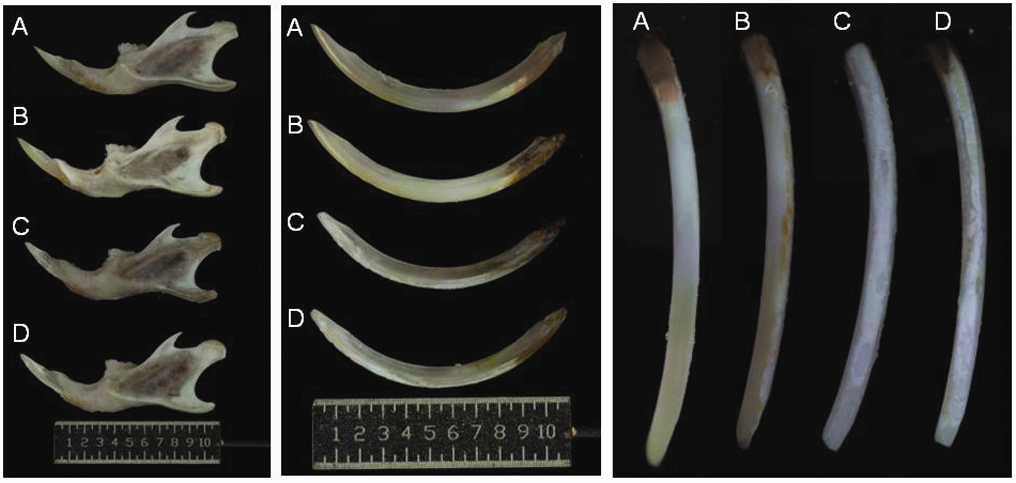


(A) *Amelx*^WT^; (B) *Amelx*^X/Y64H^; (C) *Amelx*^Y/Y64H^; (D) *Amelx*^Y64H/Y64H^ genotype groups. Scale = 11.0mm.

**Fig. S2.** *Amelx* 3D Phenotype Comparison


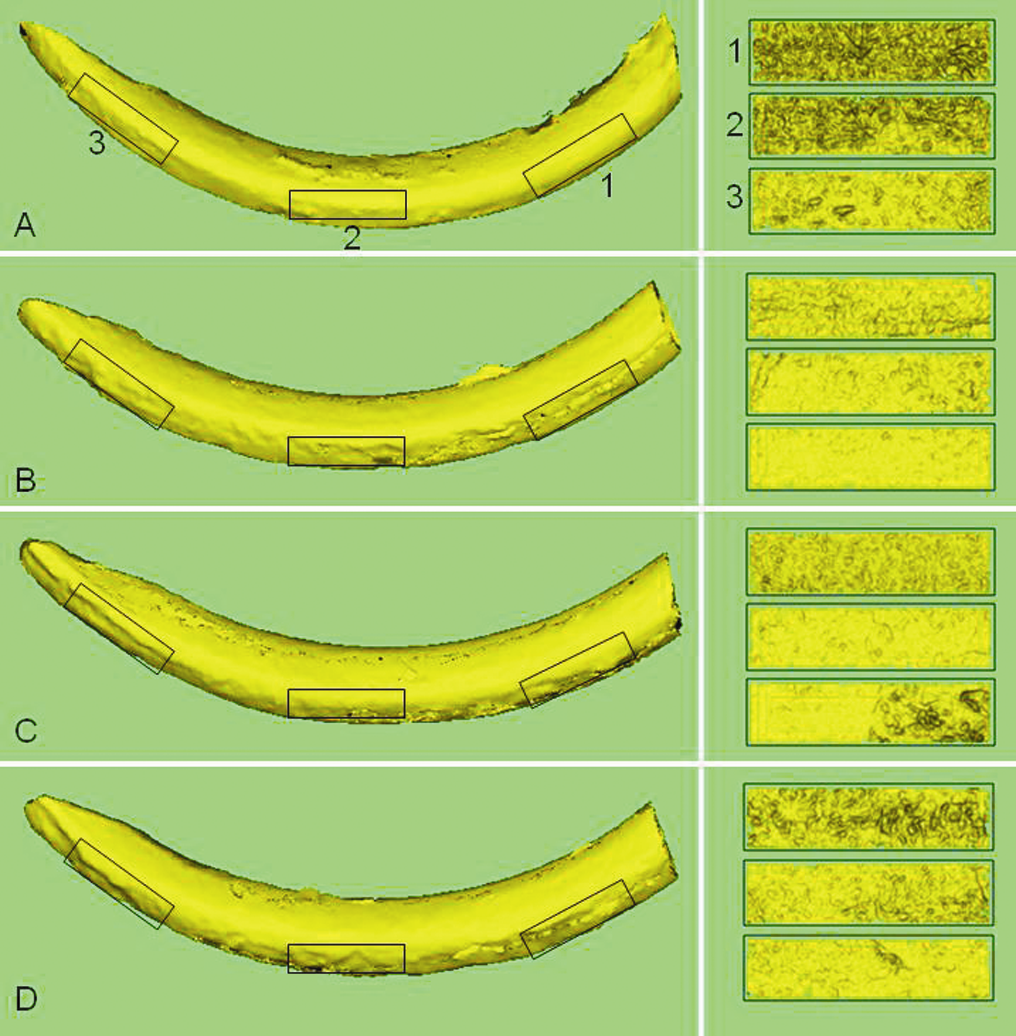
 (A) *Amelx*^WT^; (B) *Amelx*^X/Y64H^; (C) *Amelx*^Y/Y64H^; (D) *Amelx*^Y64H/Y64H^ genotype groups; (1) *cervical*; (2) *middle*; (3) *incisal* enamel surface regions/ developmental stages. No scale.

**Fig. S3.** *Enam* 2D Phenotype Comparison


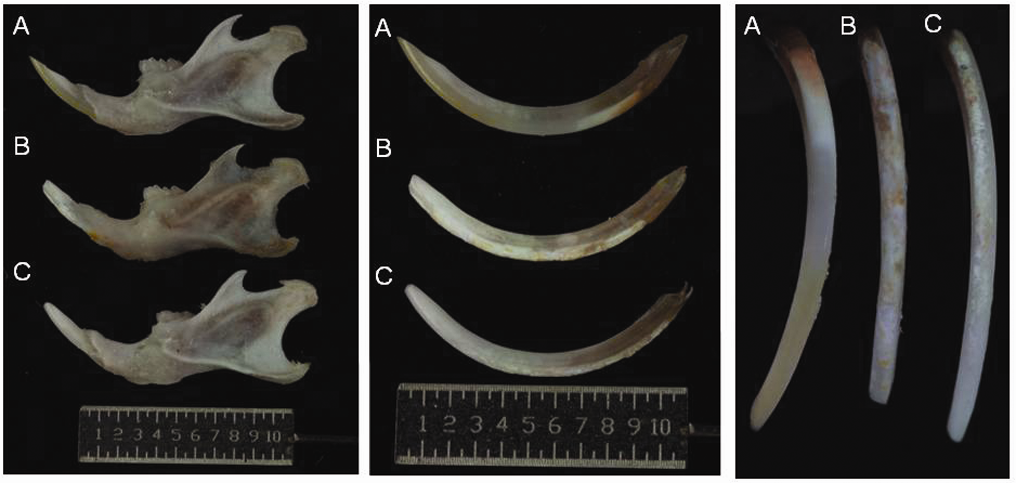


(A) *Enam wild-type*; (B) *Enam heterozygous*; (C) *Enam* *homozygous* genotype groups. Scale = 11.0mm.

**Fig. S4.** *Enam* 3D Phenotype Comparison


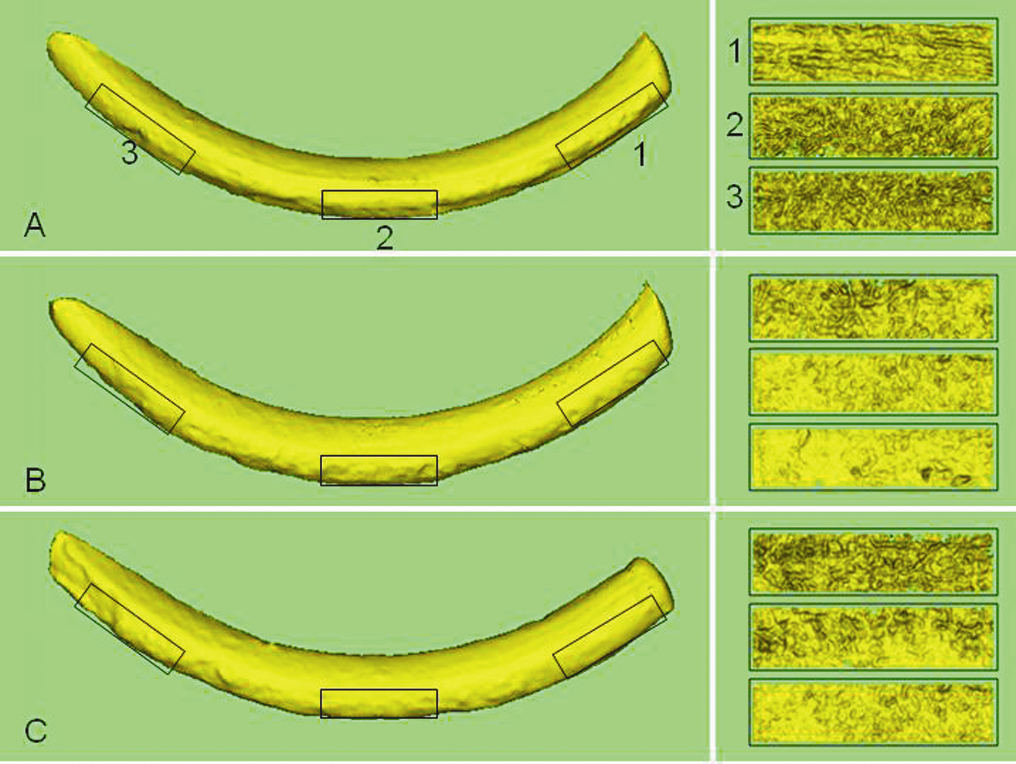


(A) *Enam*^WT^; (B) *Enam* ^Rgsc395^ *heterozygous*; (C) *Enam* ^Rgsc395^ *homozygous* genotype groups; (1) *cervical*; (2) *middle*; (3) *incisall* enamel surface regions/ developmental stages. No scale.
